# Do collagen membranes with different thickness-related mechanical properties affect the outcomes of lateral bone augmentation? An immunohistochemical analysis

**DOI:** 10.1007/s00784-026-07051-y

**Published:** 2026-08-03

**Authors:** Dongseob Lee, Young-Chang Ko, Ki-Tae Koo, Yang-Jo Seol, Obin Kwon, Yong-Moo Lee, Jungwon Lee

**Affiliations:** 1 Department of Periodontology, School of Dentistry and Dental Research Institute, Seoul National University, 101 Daehak-ro, Jongno-gu, Seoul, 03080 Republic of Korea; 2 National Dental Care Center for Persons with Special Needs, Seoul National University Dental Hospital, Seoul, Republic of Korea; 3 Department of Biochemistry and Molecular Biology, Seoul National University College of Medicine, Seoul, Korea; 4 One-Stop Specialty Center, Seoul National University Dental Hospital, Seoul, Korea

**Keywords:** Guided bone regeneration (GBR), Collagen membrane, Mechanical properties

## Abstract

**Objectives:**

This study evaluated immunohistochemical profiles associated with guided bone regeneration (GBR) using collagen membranes with different thickness-related mechanical properties to characterize membrane-related biological responses during lateral bone augmentation.

**Methods:**

Standardized mandibular defects in beagle dogs were grafted with bone substitutes and covered with one of two collagen membranes with different thickness-related mechanical properties or left uncovered. At 8 and 16 weeks, tissues were analyzed for markers associated with osteogenesis (ALP), vascular/perivascular and myofibroblast-associated responses (α-SMA), inflammation-related cell profiles (iNOS and CD206), and bone remodeling (TRAP, Cathepsin K, and collagen type I).

**Results:**

At 8 weeks, the FM group showed a trend toward higher numbers of CD206-positive cells in the coronal region. iNOS-positive cells did not differ significantly among groups in any region. However, most macrophage-related outcomes were not statistically significant. The SM group exhibited significantly greater collagen type I deposition, particularly in deeper regions, although this appeared to represent early unmineralized matrix rather than enhanced bone formation. TRAP-positive cells were more frequently observed in membrane-covered groups at both time points, mainly around biomaterials, suggesting remodeling-related activity. ALP, α-SMA, and Cathepsin K showed no significant intergroup differences. At 16 weeks, most immunohistochemical differences had diminished across all groups.

**Conclusions:**

Collagen membranes with different thickness-related mechanical properties were associated with distinct early tissue responses during GBR healing. The FM group showed trends toward increased CD206-positive cells, whereas the SM group exhibited greater early collagen type I deposition. Because most inflammation-related outcomes were not statistically significant, these findings should be interpreted cautiously and considered exploratory.

**Supplementary Information:**

The online version contains supplementary material available at 10.1007/s00784-026-07051-y.

## Introduction

Guided bone regeneration (GBR) is a widely used technique in implant dentistry that involves the use of barrier membranes to prevent the infiltration of non-osteogenic soft tissues and to provide a protected space for osteogenic cells to facilitate bone regeneration [1, 2]. Various studies have investigated different aspects of barrier membranes, including their mechanical properties, application methods, and influence on bone regeneration [3–6]. One critical factor in GBR is the physical properties of collagen membranes, such as thickness and elasticity, which may affect their ability to stabilize bone substitutes and create a favorable environment for bone formation [7].

While block bone grafts have demonstrated superior space maintenance compared to particulate bone substitutes, their clinical use is often limited due to increased patient morbidity and surgical complications [8–11]. Consequently, various techniques have been developed to enhance space maintenance when using particulate bone substitutes, including the use of screws, titanium-reinforced membranes, and titanium meshes [12–15]. More recently, soft block bone substitutes have been introduced as an alternative to rigid block graft or particle type bone substitutes, showing promising clinical outcomes in reducing surgical complexity and patient morbidity [16, 17].

Our recent research explored the influence of thickness-related physical and mechanical properties of collagen membranes on GBR outcomes by comparing thin, flexible membranes with thicker, stiffer counterparts [7]. In vitro tests evaluated hydrophilicity, enzymatic stability, and mechanical properties, while in vivo experiments in a canine model assessed bone regeneration. Mandibular defects treated with deproteinized porcine bone mineral and covered with either a thinner or thicker collagen membrane, or left uncovered were analyzed at 8 and 16 weeks. The thinner membrane showed greater wettability, faster degradation, and better conformability to the graft site, and these properties were associated with greater bone formation in the previous study, particularly in coronal, middle, and apical regions. Histomorphometric analysis at 16 weeks further demonstrated that the flexible membrane significantly improved bone regeneration, suggesting that membrane adaptability and thickness-related mechanical properties may be relevant to GBR outcomes.

Although the previous study has primarily focused on radiographic and histomorphometric assessments of GBR, the underlying biological mechanisms remain to be fully elucidated. Immunohistochemical analysis allows for a deeper understanding of bone regeneration by identifying key cellular and molecular activities involved in new bone formation, including osteogenesis-associated, vascular/perivascular, myofibroblast-associated, and inflammation-related responses [18, 19]. The evaluation of the expression of bone-related markers in augmented sites using collagen membranes with different thickness-related mechanical properties can provide further insights into the role of collagen membrane characteristics in bone regeneration.

This study aimed to characterize the immunohistochemical profiles associated with lateral bone augmentation using collagen membranes with different thickness-related mechanical properties. By analyzing markers associated with osteogenesis, vascular/perivascular and myofibroblast-associated responses, inflammation-related cell profiles, and bone remodeling, the study sought to elucidate the biological responses within augmented sites and evaluate how membrane properties influence tissue regeneration in a guided bone regeneration (GBR) model.

## Materials and methods

### Ethical statement

The experiment was approved by the Institutional Animal Care and Use Committee of Seoul National University (IACUC; approval No. SNU-230306-4-1). The manuscript was done followed by the guideline ARRIVE 2.0[1].

### Sample size calculation and experimental animals

Sample size calculation was performed using G*power (version 3.1.9.7, Autenzell, Germany). From the previous study [2], the sample size was ultimately calculated to be 6 when the standard deviation and mean differences were both set at 10%, with an alpha (α) of 5% and a power (1 − β) of 80%.

Six male beagle dogs, each 1-year-old and weighing between 12 and 14 kg, were included in the study. At the time of recruitment, all animals were in good systemic and periodontal health. The dogs were given a two-week acclimation period before the experiment. Each dog was kept in a separate indoor kennel with dimensions of 90 cm in width, 80 cm in depth, and 80 cm in height. They had unlimited access to water and were provided with a standard pellet diet.

### Timeline and surgical procedures

**Fig. 1:**
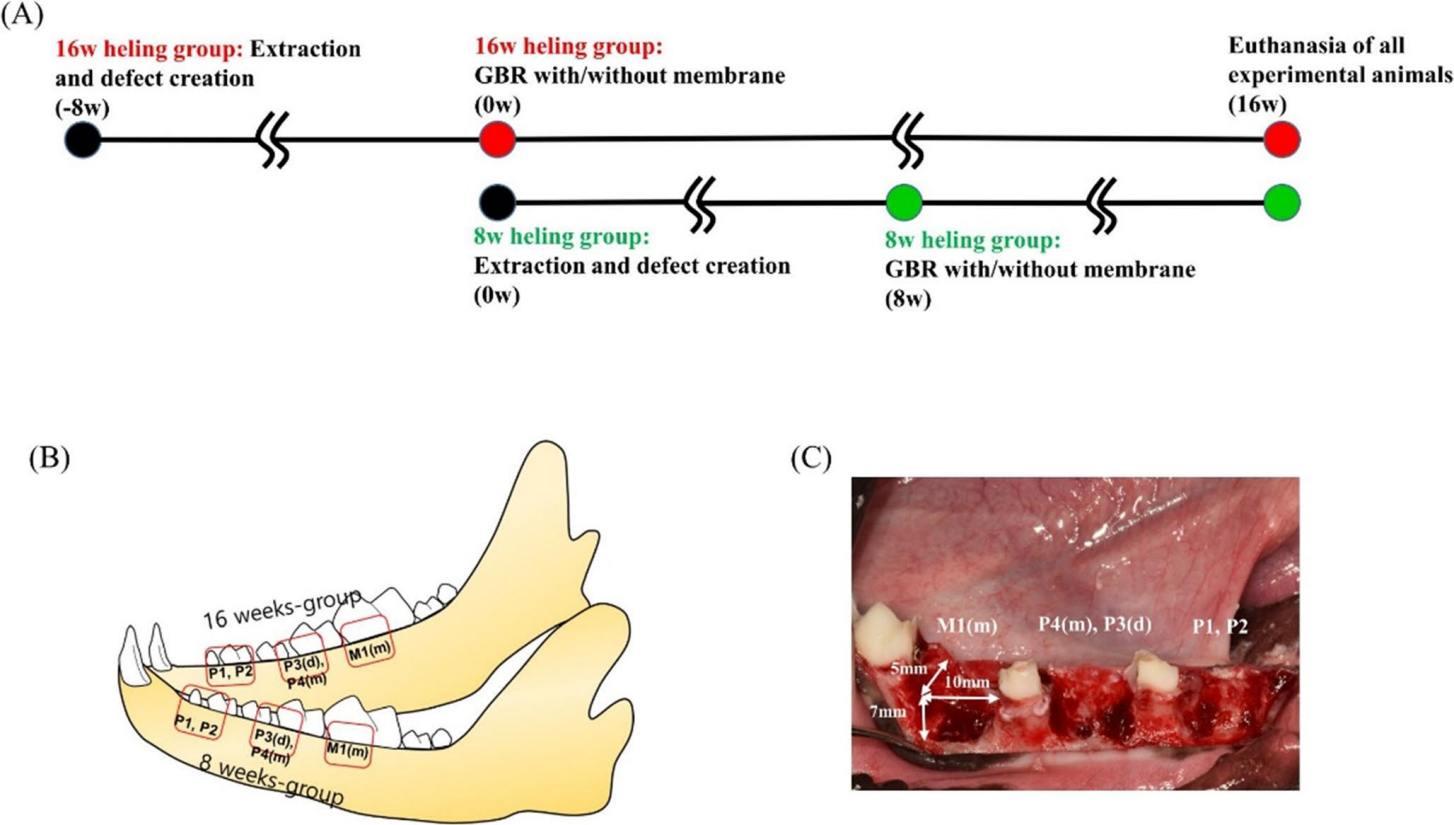
(**A**) Timeline of this study. (**B**) Experimental sites for defect creation (**C**) Defect configuration

The schematic flow of the experimental procedure is summarized in Fig. 1A.

#### Tooth extraction and defect creation

One of the randomly selected lower left or right hemimandible’s premolars and first molar were extracted. Three standardized box shape defects, measured 10 mm mesiodistally, 5 mm buccolingually, and 7 mm apicocoronally, were created on extracted teeth sites: the lower first and second premolars (P1-P2), the hemi-sectioned distal root of the third premolar and the hemi-sectioned mesial roots of the fourth premolar (P3(m)-P4(d)), and first molar (M1(m)) (Fig. 1B and C). All defect sites were allowed 8-week healing period.

#### Guided bone regeneration procedure

After 8-week healing period, each defect was randomly allocated to one of the following groups.

- Control: Bone substitutes only.
- Flexible group (FM): Bone substitutes + flexible collagen membrane (The cover flex, 25 × 30 mm, 0.3 mm thickness, Purgo, Sungnam, Gyeonggi-do, Korea).
- Stiff group (SM): Bone substitutes + stiff collagen membrane (The cover stiff, 25 × 30 mm, 0.5 mm thickness, Purgo, Sungnam, Gyeonggi-do, Korea).

Guided bone regeneration (GBR) was performed by filling the defect with deproteinized porcine bone mineral (DPBM; THE graft-cortical, Purgo, Korea), followed by application of a collagen membrane (if assigned), which was stabilized using titanium pins (Bone tack, Osstem, Korea), as previously described. Both membranes were composed of porcine type I collagen and cross-linked via the same proprietary process. As reported in our previous study [7], the difference in membrane thickness (0.3 mm vs. 0.5 mm) resulted in distinct physical properties; the stiff membrane exhibited significantly higher tensile strength and lower water wettability compared to the flexible membrane.

**Fig. 2:**
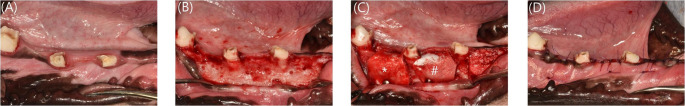
Clinical photographs when GBR performed. (**A**) Preoperative photograph (**B**) Flap elevation and decortication of the recipient site (**C**) Placement of bone substitutes, covered with either a flexible membrane (*), a stiff membrane (#), or left uncovered (**D**) Primary closure for all recipient sites

Following the surgical procedure, all animals were administered with antibiotics (Cefazolin, 20 mg/kg; Chongkundang Pharmaceutical Corp, Seoul, Korea) and analgesics (Toranzin, 5 mg/kg; Samsung Pharm., Gyeonggi-do, Korea) intravenously. For three days postoperatively, their diet was supplemented with antibiotics (Amoxicillin, 500 mg; Chongkundang Pharm., Seoul, Korea) and analgesics (Ibuprofen, 400 mg; Daewoong Pharm., Seoul, Korea). Sutures were removed 14 days after surgery. Each hemimandible was allowed to heal for either 8 or 16 weeks. All surgical procedures are depicted in Fig. 2.

### Animal euthanasia

All animals were euthanized via carotid injection of a lethal dose of potassium chloride (75 mg/kg; Jeil Pharm., Daegu, Korea). Block biopsies, including the experimental sites, were harvested for histological examinations. The excised hemimandibles were rinsed with saline and preserved in 10% buffered formaldehyde solution for 2 days.

### Histologic preparation

The tissue blocks were dissected along the bucco-lingual plane at the most central region using a diamond saw (Exakt^®^ Apparatebau, Norderstedt, Germany). The tissue sections underwent demineralization in 12.5% EDTA solution for 8 weeks, a protocol supported by previous study using canine bone samples [20]. After demineralization, the specimens were embedded in paraffin wax. Multiple paraffin-embedded specimens were sectioned into slices approximately 3 μm thick and stained with seven different antibodies for immunohistochemical analysis. Histological observations were conducted using a light microscope (DM LB; Leica Microsystems, Wetzlar, Germany), and digital images of the sections were captured with a digital camera (DC300F; Leica Microsystems, Wetzlar, Germany) and stored for further analysis.

### Histochemistry

TRAP histochemistry was performed to identify TRAP-positive multinucleated cells, which may include osteoclast-lineage cells, macrophage-lineage cells, or foreign-body giant cells, within the augmented area. After deparaffinization and rehydration, sections were incubated in a freshly prepared enzymatic reaction solution consisting of Napthol AS-MX phosphate (Sigma, N-4875) as the substrate, Fast Red Violet LB salt (Sigma, F-3381) as the diazonium coupling agent, and 50 mM sodium acetate anhydrous buffer (Sigma, S-2889; pH 5.0–5.2) for 30–40 min at room temperature. The reaction produced an insoluble red precipitate within TRAP-positive cells. Following incubation, slides were rinsed thoroughly in distilled water and lightly counterstained with hematoxylin.

### Immunohistochemistry

Immunohistochemical staining was performed according to the manufacturer’s protocol. Briefly, six different antibodies targeting Alkaline Phosphatase (ALP) (osteoblast), alpha-smooth muscle actin (α-SMA; vascular/perivascular and myofibroblast-associated marker), Cathepsin K (protease from osteoclast), inducible nitric oxide synthase (iNOS; inflammation-associated marker used as a proxy for M1-like macrophage-related responses), CD206, used as a proxy marker associated with M2-like macrophage-related responses, and collagen type I were used for immunohistochemical staining.

All primary antibodies used in this study were validated and reported with their corresponding research resource identifiers (RRIDs) to ensure methodological transparency and reproducibility: anti-ALP (RRID: AB_2854065), anti-αSMA (RRID: AB_3675461), anti-Cathepsin K (RRID: AB_3718108), anti-iNOS (RRID: AB_2152737), anti-CD206 (RRID: AB_2851091), and anti-Collagen type I (RRID: AB_2260734).

### Histological and immunohistochemical quantitative analysis

Histomorphometric analysis quantifies the structural characteristics of regenerated tissues, such as the proportion and distribution of mineralized and non-mineralized areas, whereas immunohistochemical analysis identifies and quantifies specific proteins or cell phenotypes, providing insights into the underlying biological processes of regeneration. To investigate the effects of different membrane types on bone regeneration, histological and immunohistochemical (IHC) analyses were conducted within the augmented area. As defined in a previous study [7], the augmented region was delineated as the space spanning from the mineralized tissue adjacent to the preexisting lamellar bone on the lingual and apical sides to the regenerated tissue and the dense, periosteum-like connective tissue on the buccal side. Based on the vertical distance from the alveolar crest, this area was subdivided into three regions: coronal (0–2 mm apical to the crest), middle (2–4 mm), and apical (4–6 mm). Immunohistochemical analysis of ALP, α-SMA, Cathepsin K, collagen type I, iNOS, and CD206 was performed within each of the three regions. The IHC Profiler tool [21] was used to quantify highly stained positive areas in ALP and Cathepsin K stained sections. The stained area percentage was calculated and compared regardless of staining intensity for α-SMA and collagen type I-stained sections. For iNOS and CD206, quantification was performed using ImageJ. After color deconvolution with the H-DAB vector, DAB-positive areas were isolated by manually adjusting the threshold. Positive cells were then automatically counted using the function for analyzing particle. A size filter was applied to exclude background artifacts, and three standardized regions of interest (1 × 1 mm), corresponding to the coronal, middle, and apical locations, were used for all specimens. Positively stained cells were counted, and the iNOS⁺/CD206⁺ cell ratio was calculated and analyzed. One standardized region of interest (1 × 1 mm) was selected in each vertical location (coronal, middle, and apical), and TRAP-positive cells within these ROIs were assessed.

### Statistical analysis

Data are expressed as mean ± standard deviation and median [minimum, maximum]. Group comparisons were performed using the Kruskal-Wallis test, followed by Bonferroni-adjusted post-hoc analysis. Statistical analyses were conducted using SPSS (version 25.0; IBM Corp., Armonk, NY, USA) and GraphPad Prism (version 10.0.2; GraphPad Software, San Diego, CA, USA). A p-value of less than 0.05 was considered statistically significant.

## Results

### TRAP-positive cell

**Fig. 3:**
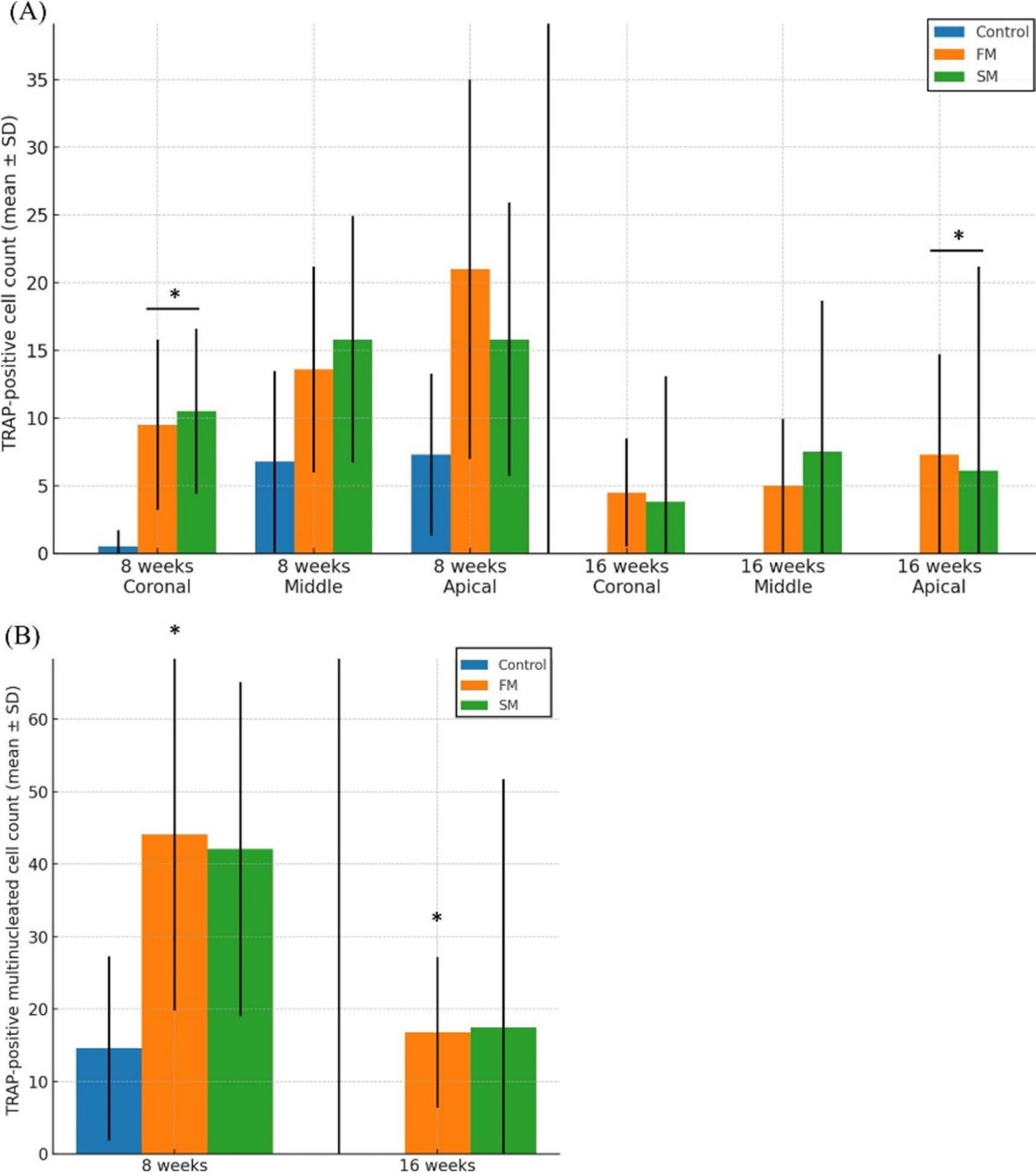
Quantitative analysis of TRAP-positive multinucleated cells. (**A**) TRAP-positive cell counts within the coronal, middle, and apical regions at 8 and 16 weeks. Data are presented as mean ± SD. (**B**) Total TRAP-positive cell counts within the whole volume of interest (VOI) at 8 and 16 weeks. Data are presented as mean ± SD. FM: flexible membrane; SM: stiff membrane. *p < 0.05 vs. Control

**Fig. 4:**
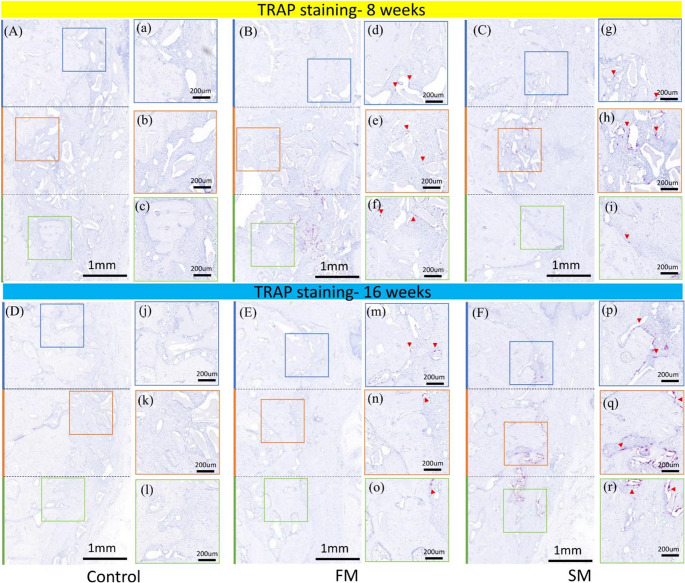
Representative images of tartrate-resistant acid phosphatase (TRAP) staining. Each specimen was delineated along the lingual pristine margin (left side of each image) and systematically divided into three regions: coronal (blue), middle (orange), and apical (green). (**A**–**C**) Control, FM, and SM groups at 8 weeks of healing; (**D**–**F**) at 16 weeks of healing. (a–r) Magnified views of the boxed areas in the corresponding images. TRAP-positive multinucleated cells are indicated by red arrowheads. Purple stained positive cells primarily localized along the bone surfaces at the interface with residual graft particles

TRAP-positive cell numbers were evaluated across the entire augmented area among the three groups. The FM and SM groups exhibited significantly higher cell numbers than the control at both 8 weeks (FM: 44.1 ± 24.3; SM: 42.1 ± 23.0; Control: 14.6 ± 12.7; *p* = 0.049) and 16 weeks (FM: 16.8 ± 10.4; SM: 17.5 ± 34.2; Control: 0.0 ± 0.0; *p* = 0.025) as shown in supplementary Tables 1 and Fig. 3. The distribution of TRAP-positive cells was assessed in the coronal, middle, and apical regions of the augmented area at both 8 and 16 weeks demonstrated in Table 1; Fig. 4. At 8 weeks, the coronal region showed significantly higher TRAP-positive cell numbers in the FM (9.5 ± 6.3) and SM (10.5 ± 6.1) groups compared to the Control group (0.5 ± 1.2) (*p* = 0.018). Although the middle and apical regions demonstrated a trend of increased TRAP-positive cells in membrane-treated groups, the differences were not statistically significant. At 16 weeks, TRAP-positive cells markedly declined across all regions in the Control group (0.0 ± 0.0), while the FM and SM groups retained detectable levels of TRAP-positive cells, particularly in the apical region (7.3 ± 7.4 in FM, 6.1 ± 15.1 in SM; *p* = 0.010).

**Table 1:** TRAP positive cell numbers in coronal, middle, and apical areas

Time	Area	Control	FM	SM	*p*-value
8weeks	Coronal	0.5 ± 1.2 0(0,3)	9.5 ± 6.3* 10.5(0,18)	10.5 ± 6.1* 13.5(0,16)	0.018
	Middle	6.8 ± 6.7 7(0,17)	13.6 ± 7.6 16(0,20)	15.8 ± 9.1 18(0,27)	0.098
	Apical	7.3 ± 6.0 9.5(0,15)	21 ± 14.0 23.5(0,36)	15.8 ± 10.1 17(0,30)	0.098
	p-value	0.08	0.20	0.27	
16weeks	Coronal	0 ± 0 0(0,0)	4.5 ± 4.0 5.0(0,9)	3.8 ± 9.3 0(0,23)	0.06
	Middle	0 ± 0 0(0,0)	5.0 ± 4.9 4.5(0,13)	7.5 ± 11.2 1(0,26)	0.07
	Apical	0 ± 0 0(0,0)	7.3 ± 7.4* 5(0,21)	6.1 ± 15.1* 0(0,37)	0.01
	p-value	1	0.84	0.49	

Values are presented as mean ± standard deviation, median(min, max)

* Statistically significant compared with Control group

### Cathepsin K staining

**Fig. 5:**
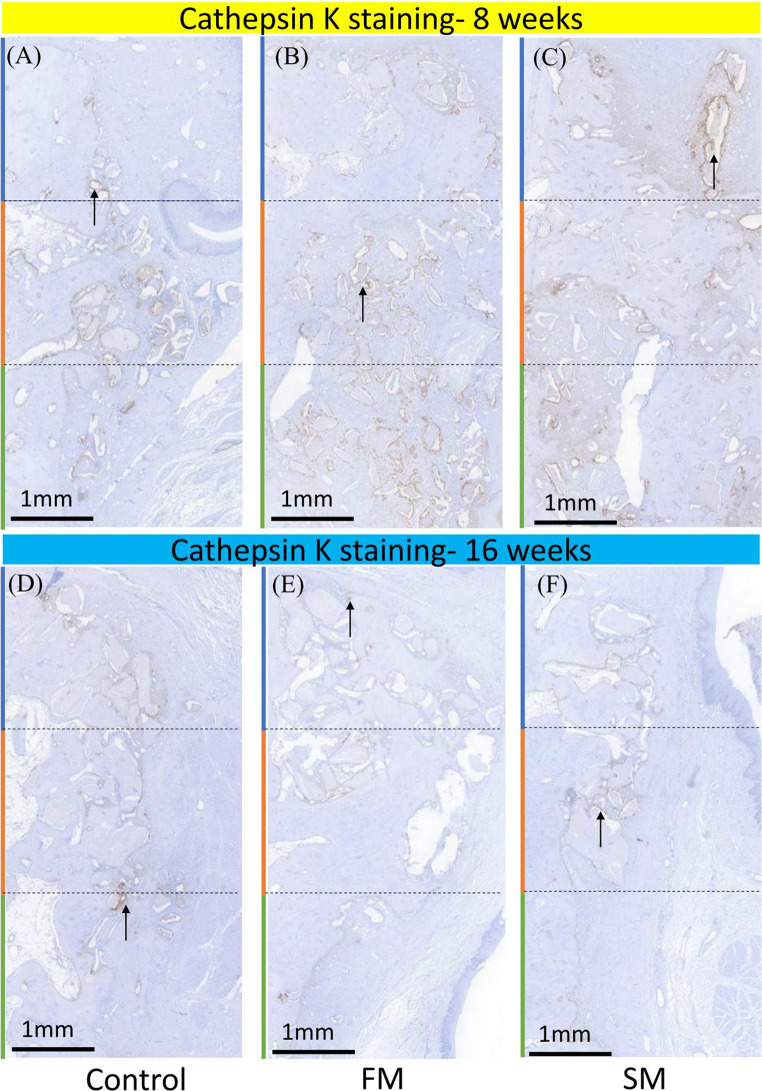
Representative images of cathepsin K staining. Specimens were aligned along the lingual pristine margin (left side of each image) and partitioned into coronal (blue), middle (orange), and apical (green) regions. (**A**–**C**) Control, FM, and SM groups at 8 weeks of healing. Areas with intense brown staining, indicative of osteoclast-associated cathepsin K expression, are indicated by black arrows; (**D**–**F**) Control, FM, and SM groups at 16 weeks of healing. Brown DAB staining indicates Cathepsin K positive osteoclasts, a protease involved in collagen degradation during bone resorption

Whole ROI analysis revealed no statistically significant differences in Cathepsin K expression among the three groups at either 8 or 16 weeks in supplementary Table 2. However, Cathepsin K staining was analyzed as shown in Table 2; Fig. 5. At 8 weeks, in coronal area, the highly positive-stained area percentage was 1.70 ± 0.35%(Control), 3.05 ± 1.61%(FM), and 3.20 ± 1.08%(SM), with significant difference (*p* = 0.022). However, no significant differences were observed among groups in the middle or apical regions at this time point. At 16 weeks, the overall trend of Cathepsin K expression remained similar, with higher mean values in the FM and SM groups. Nevertheless, statistical significance was not reached in any of the three regions.

**Table 2:** Highly positive area% per augmented area for Cathepsin K staining in coronal, middle, and apical areas

Time	Area	Control	FM	SM	*p*-value
8wk	Coronal	1.7 ± 0.3 1.6(1.1,2.2)	3.0 ± 1.6 2.5(1.4,6.1)	3.2 ± 1.0 3.1(2,4.7)	0.022*
	Middle	5.4 ± 3.4 5.2(0.5,10.6)	7.0 ± 4.2 5.8(2.6,12.5)	8.1 ± 4.9 6.0(3.2,15.7)	0.557
	Apical	2.7 ± 0.7 2.9(1.6,3.7)	3.2 ± 1.9 3.7(0.2,5.1)	3.6 ± 1.6 3.3(1.9,5.7)	0.688
	p-value	0.03 (C&M)	0.92	0.80	
16wk	Coronal	2.0 ± 1.1 1.8(0.8,4.3)	2.9 ± 1.4 2.7(1.1,5.1)	3.3 ± 1.9 3.4(0.6,6.4)	0.318
	Middle	2.5 ± 0.5 2.5(1.8,3.2)	3.5 ± 1.3 3.4(1.9,5.8)	4.0 ± 1.5 4.3(1.2,5.5)	0.09
	Apical	3.7 ± 1.5 3.2(2,6.1)	3.7 ± 2.6 3.1(1.2,8.4)	3.6 ± 1.2 3.5(2,5.6)	0.864
	p-value	0.05 (C&A)	0.71	0.56	

Values are presented as mean ± standard deviation, median(min, max)

* Statistically significant compared with Control group

### ALP-positive area

**Fig. 6:**
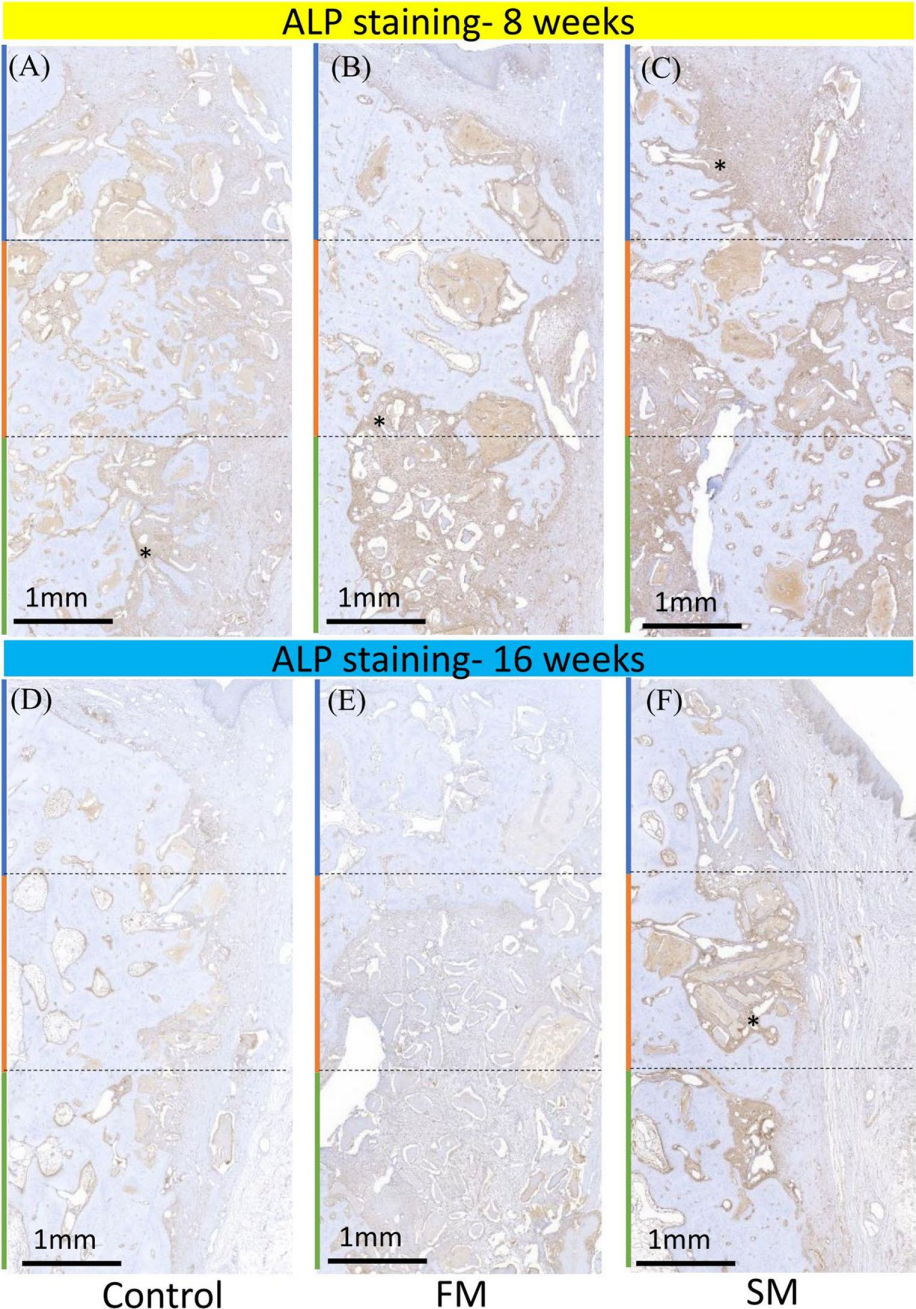
Representative images of ALP staining. Following delineation along the lingual pristine margin (located on the left side of each image), each specimen was systematically segmented into three anatomical regions: coronal (blue), middle (orange), and apical (green). (**A**–**C**) Control, FM, and SM groups at 8 weeks of healing; (**D**–**F**) Control, FM, and SM groups at 16 weeks of healing. Asterisks indicate areas exhibiting intense brown staining for ALP. Positive signals are primarily localized along the surfaces of newly formed bone adjacent to graft particles

The overall ALP-positive area percentage across the augmented volume did not differ significantly among the three groups at either time point (Supplementary Table 3). As described in Table 3; Fig. 6, although higher staining was observed in the SM group, particularly in the coronal and apical regions at 8 weeks, the differences among the three groups were not statistically significant. At 16 weeks, ALP-positive staining remained similar or slightly decreased over time, with no statistically significant differences observed between the groups at any region.

**Table 3:** Highly positive area% per augmented area for ALP staining in coronal, middle, and apical areas

Time	Area	Control	FM	SM	*p*-value
8weeks	Coronal	4.1 ± 2.5 3.6(0.5,8.3)	4.8 ± 2.9 3.8(2.4,9.8)	7.8 ± 5.9 6.2(1.7,15.4)	0.587
	Middle	5.4 ± 3.4 5.2(0.5,10.6)	7.0 ± 4.2 5.8(2.6,12.5)	8.1 ± 4.9 6.0(3.2,15.7)	0.557
	Apical	7.0 ± 2.8 7.0(3.5,10.6)	8.2 ± 4.2 7.8(4.2,13.1)	9.7 ± 5.8 8.7(2.6,18.9)	0.676
	p-value	0.24	0.24	0.80	
16weeks	Coronal	5.2 ± 3.3 4.2(1.5,10.7)	4.2 ± 2.4 4.3(0.5,7.4)	6.5 ± 4.3 6.4(2.1,11.8)	0.781
	Middle	6.6 ± 2.6 6.1(3.8,11.3)	6.3 ± 2.6 6.2(2.2,10.4)	6.5 ± 3.4 6.2(2.9,10.6)	0.960
	Apical	6.3 ± 2.9 5.6(2.7,11.3)	7.8 ± 2.4 8.0(4.7,11.4)	8.7 ± 4.8 7.3(3.9,16.8)	0.449
	p-value	0.47	0.08	0.58	

Values are presented as mean ± standard deviation, median(min, max)

### Collagen type I staining

**Fig. 7:**
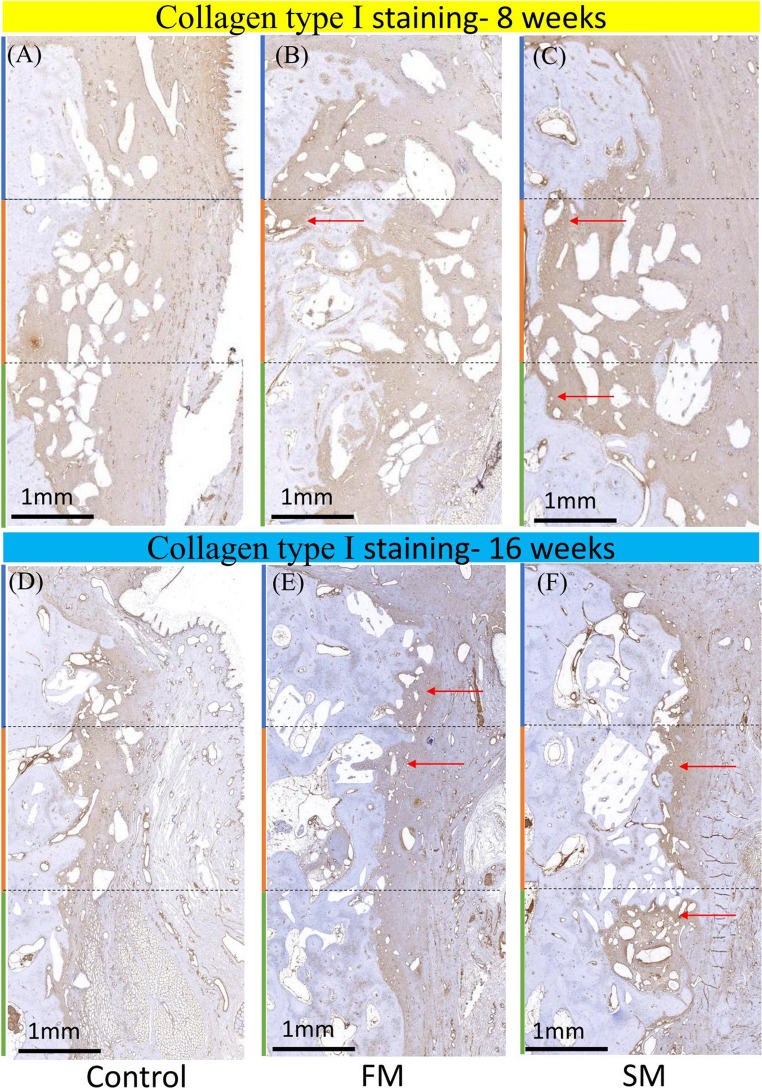
Representative images of Collagen staining. Specimens were aligned along the lingual pristine margin (left side of each image) and partitioned into coronal (blue), middle (orange), and apical (green) regions. (**A**–**C**) Control, FM, and SM groups at 8 weeks of healing; (**D**–**F**) Control, FM, and SM groups at 16 weeks of healing. Red arrows indicate areas with intense brown staining for collagen type I

In the whole ROI comparison, the collagen type I-stained area percentage was significantly higher in the SM group (17.8 ± 7.0%) than in the control group (8.2 ± 3.2%) at 8 weeks (*p* = 0.006), while no differences were observed at 16 weeks in supplementary Table 4. Collagen type I staining was performed to assess extracellular matrix formation within the regenerating bone. In Table 4; Fig. 7, the stained area percentage in the coronal region was significantly higher in the SM group (14.3 ± 5.5%) compared to the Control group (7.1 ± 2.5%) (*p* = 0.038) at 8 weeks. Similarly, in the middle and apical regions, the SM group showed higher stained area percentages (19.6 ± 7.6% and 19.3 ± 10.3%, respectively) than the Control group (9.5 ± 5.1% and 8.3 ± 3.3%), with statistical significance (*p* = 0.018 and *p* = 0.016, respectively). At 16 weeks, the stained area percentages decreased across all regions and groups, and no significant differences were observed among the groups.

**Table 4:** Collagen type I-stained area% per augmented area in coronal, middle, and apical areas

Time	Area	Control	FM	SM	*p*-value
8weeks	Coronal	7.1 ± 2.5 7(5,12)	10.1 ± 4.8 9.5(6,19)	14.3 ± 5.5* 13(9,24)	0.038
	Middle	9.5 ± 5.1 7.5(5,16)	11.3 ± 3.3 11(8,15)	19.6 ± 7.6* 16.5(13,34)	0.018
	Apical	8.3 ± 3.3 8.5(3,13)	11.5 ± 7.5 9.5(4,26)	19.3 ± 10.3* 16.5(12,40)	0.016
	p-value	0.57	0.74	0.32	
16weeks	Coronal	6.6 ± 2.3 7.5(2,8)	7.3 ± 3.5 8(3,13)	7.3 ± 4.6 6.5(2,16)	0.630
	Middle	9.3 ± 2.6 8(7,14)	8.5 ± 2.4 9(4,11)	10.5 ± 6.8 8.5(2,21)	0.981
	Apical	13.1 ± 2.4 12.5(11,17)	12.8 ± 4.6 12.5(7,19)	12.8 ± 5.6 13(5,21)	0.961
	p-value	< 0.001(C&A)	0.08	0.21	

Values are presented as mean ± standard deviation, median(min, max)

* Statistically significant compared with Control group

### α-SMA staining

**Fig. 8:**
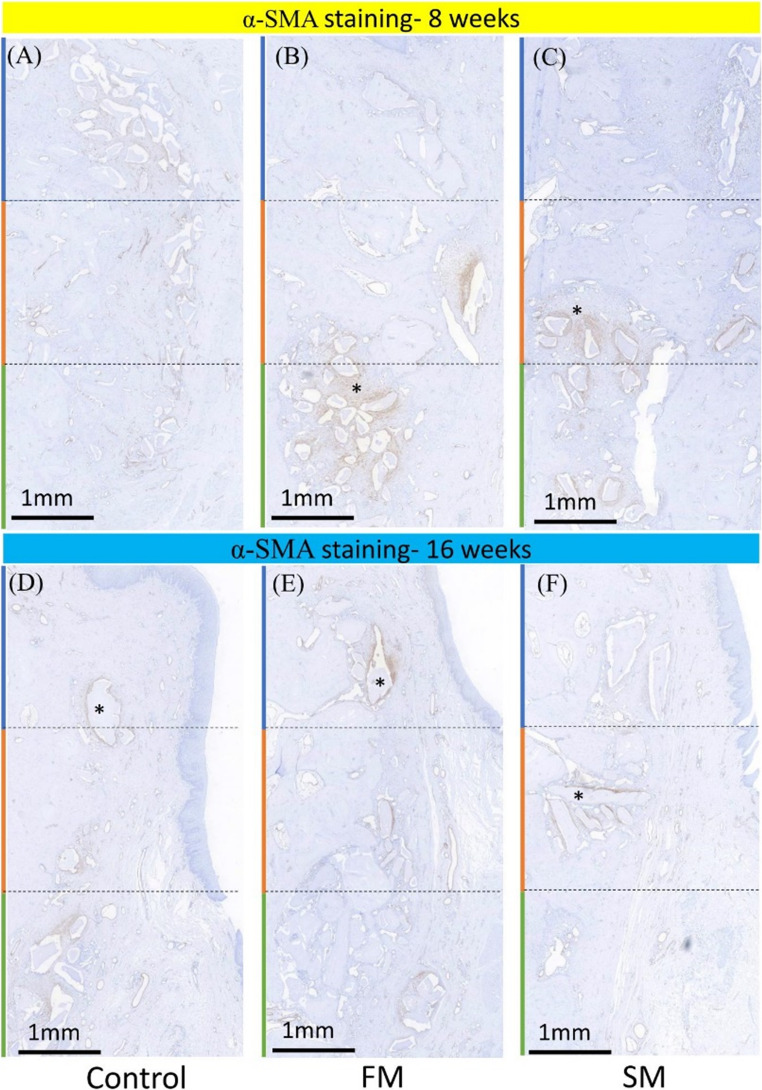
Representative images of α-SMA staining. Each specimen was delineated along the lingual pristine margin (left side of each image) and systematically segmented into three anatomical regions: coronal (blue), middle (orange), and apical (green). (**A**–**C**) Control, FM, and SM groups at 8 weeks of healing; (**D**–**F**) at 16 weeks of healing. Asterisks indicate regions with intense brown staining, indicative of α-SMA expression

The α-SMA-stained area percentage across the entire augmented area showed no significant differences among the three groups at either 8 or 16 weeks (Supplementary Table 5). In Table 5; Fig. 8, α-SMA staining percentages were comparable among the groups in all regions, showing no statistically significant differences. At 16 weeks, a mild increase in α-SMA expression was observed in the coronal region of the FM group compared to the Control and similar trends were noted in the middle and apical regions, but none of the differences reached statistical significance.

**Table 5:** α-SMA stained area percent in coronal, middle, and apical areas

Time	Area	Control	FM	SM	*p*-value
8weeks	Coronal	5.8 ± 1.5 5.8(3.8,8.5)	5.5 ± 3.1 5.4(1,9.8)	5.9 ± 5.7 4(1.4,16.9)	0.623
	Middle	6.4 ± 2.5 5.4(4.2,10.7)	4.8 ± 2.6 5.4(1.3,8)	5.4 ± 3.2 4.4(1.8,11.2)	0.641
	Apical	6.4 ± 3.6 5.5(2.2,12.1)	4.7 ± 1.7 5.2(2.4,7.1)	4.9 ± 2.9 5.8(1,8.8)	0.810
	p-value	1	0.84	1	
16weeks	Coronal	6.3 ± 3.0 5.3(3.6,12.3)	7.7 ± 3.8 7.2(2.7,13)	6.7 ± 3.2 6.8(3.2,10.7)	0.805
	Middle	7.0 ± 3.1 3.1(3.4,12.4)	7.6 ± 3.7 6.6(2.5,10.2)	6.8 ± 1.5 7.1(5,9)	0.939
	Apical	8.2 ± 4.2 8.1(3.8,14.1)	6.2 ± 2.5 6.6(1.6,9)	6.8 ± 2.4 6.1(4,10.3)	0.834
	p-value	0.91	0.83	0.88	

Values are presented as mean ± standard deviation, median(min, max)

### CD206-positive and iNOS-positive cells

**Fig. 9:**
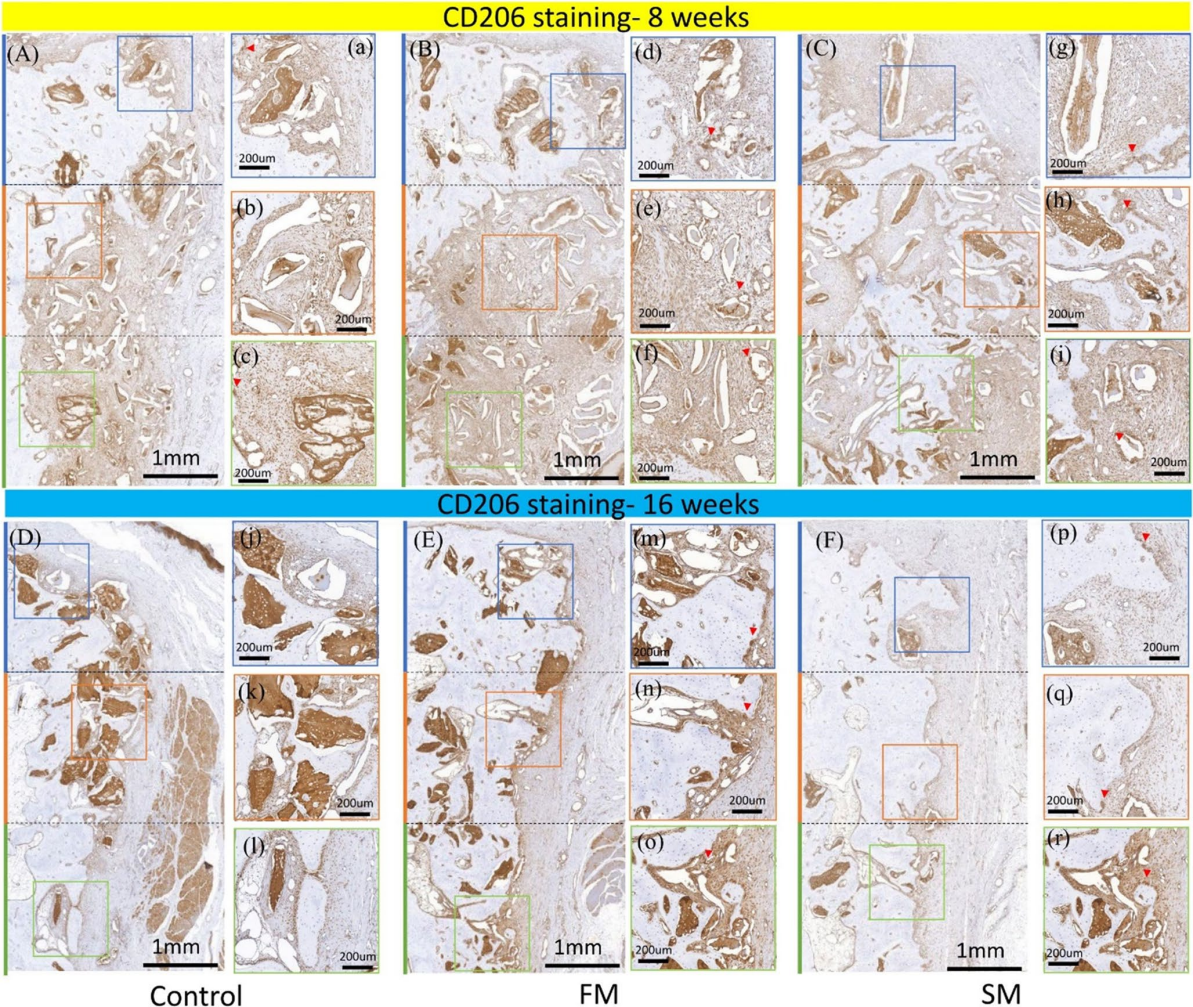
Representative images of CD206 staining. Each specimen was delineated along the lingual pristine margin (left side of each image) and systematically segmented into three anatomical regions: coronal (blue), middle (orange), and apical (green). (**A**–**C**) Control, FM, and SM groups at 8 weeks of healing; (**D**–**F**) at 16 weeks of healing. (a–r) Magnified views of the boxed areas in the corresponding images. Red arrowheads indicate CD206-positive cells, used as a proxy marker associated with M2-like macrophage-related responses

**Fig. 10:**
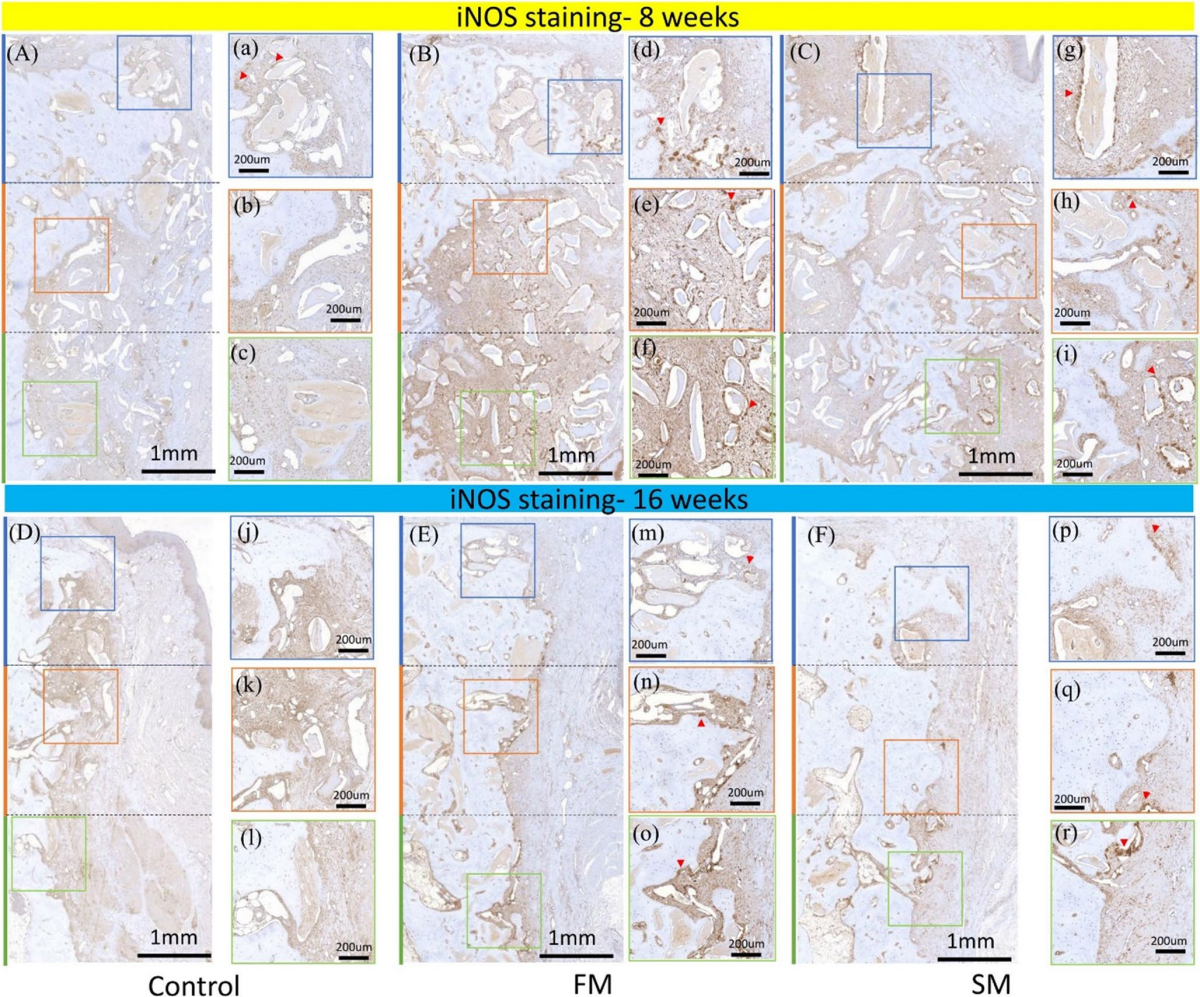
Representative images of iNOS staining. Each specimen was delineated along the lingual pristine margin (left side of each image) and systematically segmented into three anatomical regions: coronal (blue), middle (orange), and apical (green). (**A**–**C**) Control, FM, and SM groups at 8 weeks of healing; (**D**–**F**) at 16 weeks of healing. (a–r) Magnified views of the boxed areas in the corresponding images. Red arrowheads indicate iNOS-positive cells, used as a proxy marker associated with M1-like macrophage-related responses

When analyzed across the entire ROI, the numbers of CD206-positive and iNOS-positive cells did not significantly differ among groups at either time point (Supplementary Tables 6 and 7). Region-specific analysis revealed that at 8 weeks, the FM group showed a higher number of CD206-positive cells in the coronal region (52.3 ± 33.7) compared to the Control group (12.5 ± 13.7) (*p* = 0.050), while no significant differences were found in the middle and apical regions. For iNOS-positive cells, no statistically significant differences were observed in any region at either time point (Tables 6 and 7; Figs. 9 and 10). The iNOS/CD206 ratio, used as an exploratory proxy for macrophage polarization-related balance, also showed no statistically significant intergroup differences when analyzed across the entire augmented area (Supplementary Table 8). However, both the FM and SM groups exhibited consistently lower iNOS/CD206 ratios compared to the Control group at both time points (Table 8).

**Table 6:** CD206 stained numbers in coronal, middle, and apical areas

Time	Area	Control	FM	SM	*p*-value
8weeks	Coronal	12.5 ± 13.7 8.5(0,37)	52.3 ± 33.7 47.5(17,105)	31.0 ± 27.0 30(1,75)	0.05†
	Middle	40.1 ± 39.4 32(0,113)	61.0 ± 55.3 40(24,172)	28.6 ± 17.3 29.5(1,54)	0.345
	Apical	42.3 ± 42.4 32(0,114)	56.0 ± 43.0 38.5(22,135)	41.8 ± 15.3 38.5(27,66)	0.850
	p-value	0.25	0.96	0.52	
16weeks	Coronal	13.8 ± 13.0 13(0,33)	22.8 ± 13.4 25.5(6,37)	23.5 ± 9.1 22.5(12,34)	0.323
	Middle	19.6 ± 20.2 15(2,58)	35.5 ± 28.5 29.5(11,89)	23.0 ± 10.8 22.5(11,36)	0.520
	Apical	14.8 ± 12.9 9.5(8,41)	24.5 ± 19 20(4,54)	22.5 ± 10.7 17.5(14,41)	0.228
	p-value	0.80	0.67	0.92	

Values are presented as mean ± standard deviation, median(min, max)

† The Kruskal–Wallis test revealed a statistically significant difference among the groups (*p* < 0.05). However, Bonferroni-adjusted post hoc comparisons did not detect any statistically significant differences between individual group pairs

**Table 7:** iNOS stained numbers in coronal, middle, and apical areas

Time	Area	Control	FM	SM	*p*-value
8weeks	Coronal	11.0 ± 18.3 2.5(2,48)	8.3 ± 9.3 3.5(2,26)	2.1 ± 0.4 2(2,3)	0.07
	Middle	44.3 ± 94.2 2(2,236)	10.0 ± 11.8 4.5(2,32)	5.8 ± 5.1 4(2,16)	0.795
	Apical	49.5 ± 96.9 9(1,246)	10.5 ± 9.7 6.5(2,26)	7.8 ± 6.5 7(2,16)	0.886
	p-value	0.97	0.85	0.06	
16weeks	Coronal	10.1 ± 17.6 2(2,46)	9.8 ± 10.3 4.5(2,24)	17.5 ± 24.8 4(2,18)	0.782
	Middle	9.1 ± 11.0 2.5(2,27)	29.0 ± 35.6 29.5(11,89)	23.0 ± 10.8 22.5(11,36)	0.442
	Apical	15.0 ± 18.0 5(2,41)	19.1 ± 24.9 7(2,64)	13.6 ± 17.0 5.5(2,45)	0.972
	p-value	0.64	0.58	0.97	

Values are presented as mean ± standard deviation, median(min, max)

**Table 8:** iNOS/CD206 ratio in coronal, middle, and apical areas

Time	Area	Control	FM	SM	*p*-value
8weeks	Coronal	5.6 ± 9.6 0.3(0.1,24)	0.3 ± 0.5 0.1(0.04,1.5)	0.4 ± 0.7 0.1(0.03,2)	0.28
	Middle	4.5 ± 8.7 0.1(0.02,22)	0.3 ± 0.3 0.05(0.03,0.91)	1.1 ± 2.3 0.1(0.06,6)	0.58
	Apical	5.6 ± 12.0 0.1(0.07,30)	0.3 ± 0.3 0.1(0.04,0.93)	0.2 ± 0.2 0.2(0.05,0.41)	0.70
	p-value	0.57	0.80	0.65	
16weeks	Coronal	8.1 ± 18.4 0.2(0.1,46)	0.4 ± 0.2 0.4(0.05,0.75)	0.9 ± 0.3 0.3(0.06,3.5)	0.91
	Middle	2.5 ± 5.3 0.3(0.03,13.5)	0.8 ± 0.9 0.3(0.2,2.7)	0.8 ± 1.2 0.2(0.06,3.3)	0.76
	Apical	1.8 ± 2.3 0.5(0.05,5.1)	0.8 ± 0.9 0.4(0.1,2.5)	0.7 ± 0.9 0.3(0.1,2.7)	0.67
	p-value	0.87	0.94	0.86	

## Discussion

In this canine GBR model, collagen membranes with different thickness-related mechanical properties were associated with possible differences in the inflammatory and regenerative tissue milieu at 8 weeks post-surgery. The FM group showed a trend toward increased CD206-positive cells, although most immune-related outcomes were not statistically significant. The SM group showed significantly greater collagen type I deposition in the augmented area; however, this finding may reflect early unmineralized matrix or collagenous tissue rather than enhanced bone formation. Markers associated with osteogenesis, vascular/perivascular and myofibroblast-associated responses, and osteoclast-associated activity did not differ significantly among groups. Because animal-level clustering was not modelled, these findings should be interpreted as exploratory and hypothesis-generating rather than confirmatory.

Both membrane-covered groups showed more TRAP-positive cells than the no-membrane covered group (control), especially in the coronal area at 8 weeks. Although TRAP traditionally marks osteoclasts involved in bone resorption, it is also expressed by other macrophages [22–24]. TRAP-positive cells declined by 16 weeks but remained more prominent in membrane sites, particularly deeper regions. Notably, cathepsin K, specific to active osteoclasts, did not differ among groups, implying many TRAP-positive cells surrounding the bone graft materials might not be classical osteoclasts. Considering that the TRAP-positive cells were predominantly observed surrounding the soft tissue areas adjacent to the biomaterials, it is unlikely that these represent osteoclasts typically found within bone tissue. Instead, they may represent macrophage-lineage cells or foreign-body giant cells responding to the membrane or bone graft materials [25, 26]. These cells are commonly seen around biomaterials and contribute to membrane degradation and tissue remodeling rather than direct bone resorption [25, 26]. The control group, lacking a membrane or substantial new bone, showed minimal TRAP activity. Thus, elevated TRAP-positive cells under membranes may reflect an early inflammatory or remodeling response possibly related to biomaterial degradation, although causality cannot be determined from the present data. TRAP-positive cell numbers decreased by 16 weeks, which may be compatible with a transient remodeling response rather than persistent inflammation. Overall, TRAP activity in membrane-covered sites may reflect ongoing remodeling processes; however, the specific cellular contributors cannot be determined from the present data. Future studies using lineage-specific co-staining approaches could further clarify cell identity. Therefore, the identity and functional role of the TRAP-positive cells observed in the present study should be interpreted with caution.

At 8 weeks, the SM group showed significantly higher type I collagen immunostaining which was almost double that of the flexible membrane in middle and apical areas. While type I collagen is essential to the bone matrix, elevated levels at this stage may indicate unmineralized osteoid or fibrous tissue rather than mature mineralized bone [27, 28]. The stiff, cross-linked membrane, due to slower resorption and reduced permeability, may have contributed to the formation of a dense collagenous layer at the interface, resembling the formation of a pseudo-periosteum often observed with non-resorbable barrier membranes [29]. This phenomenon might be attributed to the less permeable and resorbable membranes that can delay early tissue penetration and lead to fibrous encapsulation. In contrast, the lower collagen I in the FM group may be compatible with a different pattern of matrix remodeling, although mineralization was not directly assessed in the present immunohistochemical analysis. This tendency is consistent with our previous histomorphometric analysis, which demonstrated a significantly greater mineralized area in the FM group compared with other groups [7]. By 16 weeks, group differences in collagen I had resolved, indicating that the stiff membrane’s early collagen accumulation was transient. Importantly, more collagen at 8 weeks did not correlate with better bone outcomes. Previous studies using the same model showed greater bone volume in the FM group by 16 weeks [7]. This raises the possibility that the thicker membrane was associated with a transient delay in matrix maturation, although this interpretation remains speculative. Overall, collagen deposition must be balanced for proper bone regeneration. Early matrix formation is essential; however, prolonged unmineralized collagen could theoretically be less favorable for bone regeneration, although this was not directly demonstrated in the present study. The FM group may have shown a different balance of collagen deposition and tissue remodeling by 8 weeks, but further studies correlating immunohistochemical findings with mineralized tissue formation are required.

At 8 weeks, neither ALP nor α-SMA expression significantly differed among groups, suggesting broadly comparable osteogenesis-associated and vascular/perivascular or myofibroblast-associated staining patterns regardless of membrane type. ALP, a marker of matrix formation and early mineralization [30], was evenly distributed across all conditions, suggesting broadly comparable osteogenesis-associated activity among the groups. Since ALP expression typically peaks during early healing and tapers as mineralization progresses, any differences due to membrane characteristics may have occurred earlier, possibly within the first 4 weeks [31]. Furthermore, analysis across the entire augmented area (coronal to apical) may have averaged out localized variations. Similarly, comparable α-SMA staining patterns may suggest similar vascular/perivascular and myofibroblast-associated tissue responses by 8 weeks. As α-SMA labels perivascular cells and myofibroblasts [32], this pattern may be compatible with vascular/perivascular tissue organization without clear evidence of excessive myofibroblast-associated staining, such as excessive myofibroblast activation. Both collagen membranes despite differences in stiffness and degradation showed comparable α-SMA staining patterns, which may be compatible with similar vascular/perivascular tissue organization among the groups, although vascular infiltration was not directly assessed. These findings align with prior reports showing minimal differences in vascular/perivascular staining patterns between crosslinked and non-crosslinked membranes at early time points [33]. Together, the similar ALP and α-SMA profiles suggest that fundamental regenerative processes were underway in all groups by 8 weeks. Nevertheless, because α-SMA is not specific to angiogenesis and may also be expressed by smooth muscle cells, pericytes, and myofibroblasts, interpretations regarding vascularization or angiogenesis based on α-SMA staining alone should be made with caution.

At 8 weeks, the FM group showed a trend toward increased CD206-positive cells, whereas iNOS-positive cells did not differ significantly among groups. CD206 is commonly used as a proxy marker associated with M2-like macrophage-related reparative responses, whereas iNOS is commonly used as a proxy marker associated with M1-like inflammatory responses [34]. The FM group’s hydrophilicity and better tissue adaptation may have enhanced early protein adsorption and cell infiltration, which could potentially influence macrophage responses [7]. These observations may be consistent with concepts proposed in osteoimmunology literature; however, the present data do not directly demonstrate immunomodulation or the underlying cellular signaling pathways [35].

Several mechanisms have been proposed in the literature regarding how collagen membranes may interact with immune cells. First, previous studies have suggested that protein adsorption onto collagen surfaces may influence monocyte and macrophage behavior; however, protein adsorption and integrin-related signaling were not directly assessed in the present study [36]. Second, features such as membrane porosity and wettability have been reported to facilitate cell recruitment, permitting infiltration of monocytes, macrophages, and progenitor cells that may participate in immune regulation and osteogenic activity [37]. Third, enhanced immune cell infiltration, particularly of macrophage populations with reparative marker expression and dendritic cells, may help facilitate inflammation resolution and support a regenerative environment conducive to bone formation [38]. A tendency toward higher CD206-positive cells was observed in the FM group; however, the biological significance of this finding remains uncertain because most macrophage-related outcomes were not statistically significant, potentially reflecting conditions conducive to scaffold remodeling and osteoprogenitor recruitment [39]. A timely shift from inflammatory to reparative macrophage-related responses has been proposed to contribute to bone regeneration; however, CD206 and iNOS alone cannot definitively define macrophage phenotypes. The trend toward increased CD206-positive cells in the FM group may be compatible with a regenerative healing response [34, 40]. The lack of statistically significant differences in most inflammation-related outcomes may be partly attributable to the conservative Bonferroni correction and the limited sample size inherent to small animal studies. Furthermore, emerging literature has highlighted the limitations of the binary M1/M2 classification, suggesting that macrophage phenotypes exist on a spectrum rather than in discrete states. Therefore, although a trend toward increased CD206-positive cells was observed in the FM group, these findings should be interpreted cautiously. These pathways, however, were not directly assessed in the present study and should be regarded as hypothesis-generating explanations rather than demonstrated mechanisms.

While higher mechanical strength is often regarded as advantageous in resisting soft tissue collapse in GBR procedure, this study highlights that mechanical rigidity alone may not ensure optimal regenerative outcomes. In our preclinical model, the pressure exerted by soft tissues was limited, and host–material interactions may have contributed to the observed tissue-response patterns. The stiff membrane, despite its space-maintaining potential, exhibited greater early collagen deposition and lower CD206-positive cell counts than the FM group, although most immune-related differences were not statistically significant. In contrast, the thinner and more flexible membrane showed better tissue adaptation and faster degradation, enabling early cell recruitment and constructive tissue remodeling. These advantages appeared to compensate for its lower mechanical strength in the context of small-volume augmentation. Thus, while rigid membranes such as titanium meshes are useful in large or pressure-prone defects, the biological performance of collagen membranes and their interactions with host tissues should not be underestimated. A balance between mechanical stability and biological responsiveness is essential when selecting membranes for GBR applications. Clinically, selecting a membrane that is more hydrophilic and conformable may support favorable tissue integration and remodeling. These findings suggest that barrier membranes may influence tissue responses beyond their traditional barrier function [41]. Future investigations using transcriptomic or proteomic profiling may elucidate how these early host-material interactions govern long-term regenerative outcomes [42].

However, this study has several limitations. First, as a preclinical study using a limited number of animals, statistical power is constrained. Several findings, including the trends observed in CD206 and iNOS expression, did not reach significance in pairwise comparisons after Bonferroni correction and should therefore be interpreted with caution. Second, only two post-surgical time points (8 and 16 weeks) were evaluated. Earlier inflammatory phases that are critical for macrophage polarization and early osteogenic events were not captured, and future studies incorporating earlier sampling points are warranted. Third, the limited set of immunohistochemical markers may contribute to ambiguity in the interpretation of cellular phenotypes. For example, the use of TRAP as a marker expressed by both osteoclasts and macrophage-lineage cells complicates precise identification of the cell types involved in biomaterial remodeling. Co-staining with lineage-specific markers such as CD68 or CD163 would help clarify these roles. Fourth, although the membranes were identical in composition and manufacturing, the intended difference in flexibility was achieved through variation in thickness (0.3 mm vs. 0.5 mm). Thus, the observed differences may reflect combined effects of mechanical behavior and physical dimensions rather than flexibility alone. Fifth, each defect site was treated as an independent experimental unit, and within-animal clustering effects could not be fully excluded; statistical nesting was not applied due to the limited sample size. Consequently, the reported p-values may overestimate statistical significance, and the findings should therefore be interpreted as exploratory rather than confirmatory. Future studies with larger sample sizes should incorporate hierarchical or mixed-effects statistical models that account for animal-level clustering. Sixth, no functional assessments of bone regeneration were performed such as bone volume, mineral density, or biomechanical strength. Consequently, the immunohistochemical findings cannot be directly correlated with quantitative measures of bone quality or regenerative capacity. Finally, although a single titanium pin was used for membrane fixation, which may not provide maximal mechanical stability, the limited defect size and close membrane adaptation likely minimized displacement. Nevertheless, the use of multiple fixation points may enhance reproducibility and should be considered in future studies.

In conclusion, within the limitations of this exploratory preclinical study, collagen membranes with different thickness-related mechanical properties were associated with possible differences in early tissue responses during GBR healing. The FM group showed a trend toward increased CD206-positive cells, whereas the SM group was associated with greater early collagen type I deposition. However, because multiple defects were obtained from the same animals and animal-level clustering was not modelled, the reported p-values should be interpreted with caution. These findings should therefore be considered hypothesis-generating rather than confirmatory. Further studies using larger sample sizes, animal-level statistical models, and additional lineage-specific markers are needed to clarify how membrane physical and mechanical properties influence GBR healing.

## Supplementary Information

Below is the link to the electronic supplementary material.

Supplementary Material 1

## Data Availability

The data that supports the findings of this study are available from the corresponding authors upon request.
