# Supplementary material for: Do collagen membranes with different thickness-related mechanical properties affect the outcomes of lateral bone augmentation? An immunohistochemical analysis
Source: Clin Oral Investig. 2026 Aug 3;30(8):370. doi: 10.1007/s00784-026-07051-y (PMC13433543; doi:10.1007/s00784-026-07051-y)
Supplement: Supplementary file 1 [file 784_2026_7051_MOESM1_ESM.docx]

Supplementary Table 1. Comparison of TRAP positive cell numbers among three groups.

| Time | Control | FM | SM | p-value |
| --- | --- | --- | --- | --- |
| 8weeks | 14.6±12.7  17(0,32) | 44.1±24.3* 52.5(0,69) | 42.1±23.0 43.5(0,64) | 0.049 |
| 16weeks | 0±0  0(0,0) | 16.8±10.4* 19.0(0,27) | 17.5±34.2 1(0,86) | 0.025 |

Supplementary Table 2. Comparison of highly positive area% per augmented area for Cathepsin K staining among three groups.

| Time | Control | FM | SM | p-value |
| --- | --- | --- | --- | --- |
| 8weeks | 7.6±1.7  8.1(4.5,9.5) | 9.4±3.1 9.3(4.8,13.4) | 10.4±4.0 9.6(5.8,15.5) | 0.402 |
| 16weeks | 8.3±2.7  7.9(5.0,13.4) | 10.2±4.7 8.9(5.3,18.0) | 11.1±3.7 10.7(5.1,16.2) | 0.318 |

Supplementary Table 3. Comparison of highly positive area% per augmented area among three groups.

| Time | Control | FM | SM | p-value |
| --- | --- | --- | --- | --- |
| 8weeks | 14.7±8.2  13.2(5.5,29.6) | 20.1±11.2 17.2(9.5,34.6) | 25.7±16.3 19.8(8.5,49.5) | 0.441 |
| 16weeks | 18.2±8.5  14.6(11.1,33.3) | 18.4±6.6 19.2(7.5,27.2) | 21.9±12.1 19.6(10.3,36.9) | 0.911 |

Supplementary Table 4. Comparison of collagen type I-stained area% per augmented area among three groups.

| Time | Control | FM | SM | p-value |
| --- | --- | --- | --- | --- |
| 8weeks | 8.2±3.2  7.0(5,12) | 11.1±4.8 8.9(8,20) | 17.8±7.0 16.1(13,32) | 0.006 |
| 16weeks | 9.4±1.1  9.1(9,11) | 9.3±2.5 10.2(5,12) | 10.1±5.0 9.3(3,16) | 0.796 |

| Time | Control | FM | SM | p-value |
| --- | --- | --- | --- | --- |
| 8weeks | 6.2±2.4  5.5(3.4,9.6) | 5.0±2.2 6.1(1.5,6.8) | 5.4±3.8 4.7(1.4,12.3) | 0.653 |
| 16weeks | 7.1±3.3  7.0(3.6,12.9) | 7.2±3.2 6.8(2.3,11.5) | 6.8±1.9 7.1(4,9.3) | 0.994 |

Supplementary Table 5. Comparison of α-SMA stained area percent among three groups.

| Time | Control | FM | SM | p-value |
| --- | --- | --- | --- | --- |
| 8weeks | 95.3±91.5  77.5(2,264) | 169.3±122.6 135.5(82,412) | 101.5±40.4 91.5(57,154) | 0.182 |
| 16weeks | 48.3±37.0  42.0(11,113) | 74.3±52.3 70.5(17,132) | 69±17.4 74.0(38,88) | 0.436 |

Supplementary Table 6. Comparison of CD206 stained cell numbers among three groups.

Supplementary Table 7. Comparison of iNOS stained cell numbers among three groups.

| Time | Control | FM | SM | p-value |
| --- | --- | --- | --- | --- |
| 8weeks | 104.8±209.3  13(6,530) | 28.8±27.0 18(8,77) | 15.8±9.0 14(6,29) | 0.725 |
| 16weeks | 34.3±32.5  26.5(6,80) | 62.1±63.4 45.5(6,175) | 57.6±87.2 10.5(6,225) | 0.712 |

Supplementary Table 8. Comparisons of iNOS/CD206 ratio among three groups.

| Time | Control | FM | SM | p-value |
| --- | --- | --- | --- | --- |
| 8weeks | 5.9±12.1  0.2(0.1,30.5) | 0.3±0.3  0.1(0.1,0.9) | 0.2±0.1 0.2(0,0.5) | 0.548 |
| 16weeks | 1.6±2.7  0.7(0.1,7.2) | 1.0±0.9  0.5(0.2,2.6) | 0.8±1.2 0.2(0.1,3.2) | 0.676 |
